# Mapping the Ischemic Continuum: Dynamic Multi-Omic Biomarker and AI for Personalized Stroke Care

**DOI:** 10.3390/ijms27010502

**Published:** 2026-01-03

**Authors:** Valentin Titus Grigorean, Cosmin Pantu, Alexandru Breazu, Stefan Oprea, Octavian Munteanu, Mugurel Petrinel Radoi, Carmen Giuglea, Andrei Marin

**Affiliations:** 1 Faculty of General Medicine, “Carol Davila” University of Medicine and Pharmacy, 050474 Bucharest, Romania; 2 Department of Anatomy, “Carol Davila” University of Medicine and Pharmacy, 050474 Bucharest, Romania; 3 Puls Med Association, 051885 Bucharest, Romania; 4 Department of Vascular Neurosurgery, National Institute of Neurology and Neurovascular Diseases, 077160 Bucharest, Romania; 5 Department of Neurosurgery, “Carol Davila” University of Medicine and Pharmacy, 050474 Bucharest, Romania

**Keywords:** stroke biomarkers, multi-omics integration, extracellular vesicles, cerebrospinal fluid diagnostics, single-cell transcriptomics, neuroinflammation, precision medicine, artificial intelligence, systems biology, neuroplasticity

## Abstract

Although there have been advancements in stroke treatment (reperfusion) therapy, and it has been shown that many individuals continue to suffer from partial recoveries and continuing decline in their neurological status as a result of suffering a stroke, a primary barrier to providing precise care to patients with stroke continues to be the inability to capture changes in molecular and cellular programs over time and in biological compartments. This review synthesizes evidence that represents the entire continuum of ischemia, beginning with acute metabolic failure and excitotoxicity, and ending with immune response in the nervous system, reprogramming of glial cells, remodeling of vessels, and plasticity at the level of networks, and organizes this evidence in a temporal framework that includes three biological compartments:central nervous system tissue, cerebrospinal fluid, and peripheral blood. Additionally, this review discusses new technologies which enable researchers to discover biomarkers at an extremely high resolution, including single-cell and spatial multi-omics, profiling of extracellular vesicles, proteoform-resolved proteomics, and glymphatic imaging, as well as new computational methods and machine-learning algorithms to integrate data from multiple modalities and predict trajectories of disease progression. The final section of this review will provide an overview of translationally relevant and ethically relevant issues regarding the deployment of predictive biomarkers, such as privacy, access, equity, and fairness, and emphasize the importance of global coordination of research efforts in order to ensure the clinical applicability and global equity of biomarker-based diagnostics and treatments.

## 1. Introduction—Rethinking Stroke Biomarkers as Dynamic Biological Signatures

Stroke has been at the forefront of challenges in neuroscience and global health, where stroke accounts for over 140 million disability-adjusted life years (DALYs). A growing number of strokes are caused by age-related diseases, as well as by the cumulative effects of vascular risk factors. There has been significant progress in the treatment of ischemic stroke through the use of reperfusion therapies (thrombolysis and thrombectomy); however, the majority of patients do not recover to pre-morbid levels of functioning and continue to be at risk for additional vascular events and progressive cognitive decline [1]. The lack of recovery and continued vulnerability to recurrent events and further cognitive decline suggest that ischemic stroke is not simply an isolated, transient occlusive event that can be addressed by a single intervention; instead, ischemic stroke represents an evolving, multi-scale disorder in which highly interconnected molecular, cellular, vascular, and systemic programs unfold in time. There has been a substantial increase in the clinical biomarker strategies available to evaluate stroke; however, there is a limitation to the impact of these strategies in terms of their translational potential. The current biomarker paradigm is based on measuring biomarkers at a single point in time and treating them as compartmentalized signals, thus providing a very limited view of the dynamic cerebrovascular pathology associated with stroke [2].

While some established biomarkers (such as NSE, S100B, and CRP) are used in evaluating both the diagnosis and prognosis of stroke, they also represent very limited aspects of the overall cascade of events associated with stroke and typically are not interpreted either longitudinally or mechanistically [3]. Newer biomarker types (circulating microRNAs, cargo contained within exosomes, and metabolic signatures) are also often analyzed at a single point in time, rather than as a group of temporally coordinated signals exchanged between the brain parenchyma, cerebrospinal fluid (CSF), blood, and the periphery [4].

Therefore, a more explanatory model is needed in which biomarkers are considered as time-dependent molecular states encoding the changes in injury and recovery over time. Stroke biology can be viewed as four to five overlapping phases (“phase 0–4”), each of which is driven by different, yet interrelated molecular programs [5]. During the hyperacute period (<30 min after arterial occlusion), rapid ATP depletion, ionic imbalance, and glutamate excitotoxicity drive the activation of calpain and caspases and lead to necrotic and apoptotic injury [6]. Following this initial injury, the release of DAMPs (such as HMGB1, S100A8/A9, extracellular ATP, mitochondrial DNA, and heat-shock proteins) initiates an inflammatory response through the engagement of PRRs (such as TLR2, TLR4, and RAGE), leading to downstream inflammatory signaling (via NF-kB, MAPK, and IRF) [7]. Over several hours to days, the acute phase is influenced by the activation of inflammasomes (such as NLRP3, AIM2, and NLRC4), the recruitment of leukocytes (such as CXCL1 and CCL2), and the breakdown of the ECM (through MMP-2 and MMP-9), all of which contribute to exacerbating the injury, while simultaneously beginning to establish early repair and remodeling [8,9].

During the subacute phase (several days to weeks post-injury), reprogramming of astrocytes (via STAT3/SOX9) and the formation of glial scars (via CSPGs) stabilize the microenvironment surrounding the lesion; however, this also limits structural repair [10], while microglia begin to transition from pro-inflammatory phenotypes (characterized by the expression of IL-1β and TNF-α) to reparative phenotypes (characterized by the expression of TREM2, IGF-1, and YM1), allowing for the removal of debris and the reorganization of synapses, concurrently with angiogenesis (via VEGF-A, Ang1/2, and DLL4–Notch) and neurogenesis (via SDF-1α/CXCR4) from the subventricular zone (SVZ) [11].

Finally, during the chronic phase (weeks to months after ischemic injury), system-level organization becomes the dominant feature of stroke recovery: axonal sprouting programs (via GAP-43, SPRR1A, and βIII-tubulin), the regulation of synaptic stabilization (via BDNF and NGF), and remyelination through hierarchical oligodendrocyte differentiation (initiated by Olig2 and PDGF-AA) occur alongside maladaptive persistence of inflammatory mechanisms (extended inflammasome activity, complement-mediated synaptic remodeling, and SASP) that contribute to secondary neurodegeneration and cognitive decline [12,13]. These phase transitions are encoded by coordinated molecular changes that generate temporal biomarker signatures, reflecting not only the extent of injury but the degree of adaptation and failure of recovery [14,15]; examples include cytokine polarization from TNF-α/IL-1β to IL-10/TGF-β; EV cargo evolution from miR-155 and gasdermin D to miR-124 and IGF-1; and metabolic reconfiguration from succinate-mediated HIF-1α signaling to itaconate-mediated Nrf2 activation, together defining mechanistic turning points and actionable therapeutic windows [16]. It is essential to note that these biomarker states are not confined to a single compartment, because stroke represents a disruption in the communication between the brain vasculature immune system and peripheral organs: BBB dysfunction allows for the bidirectional trafficking of CNS-derived molecules/vesicles and systemic immune cells, while glymphatic and meningeal lymphatic clearance influence metabolite and antigen dynamics, immune priming, and recovery kinetics [17]. Recent advancements in technology have made possible the development of a multi-layered, integrated biomarker approach: high-throughput transcriptomics, proteomics, lipidomics, and metabolomics allow for the resolution of coordinated network states; spatial and single-cell omics allow for region and cell type-specific post-ischemic remodeling signature; cell-free nucleic acid and EV “liquid biopsy” provide non-invasive access to the central pathobiology; and molecular PET, DTI, and perfusion MRI contextualize molecular dynamics within vascular, structural, and network architectural features, allowing for AI-based integration into composite biomarker states with predictive value greater than single marker approaches for infarct progression, hemorrhage risk, and cognitive trajectory [18,19].

**Figure 1:**
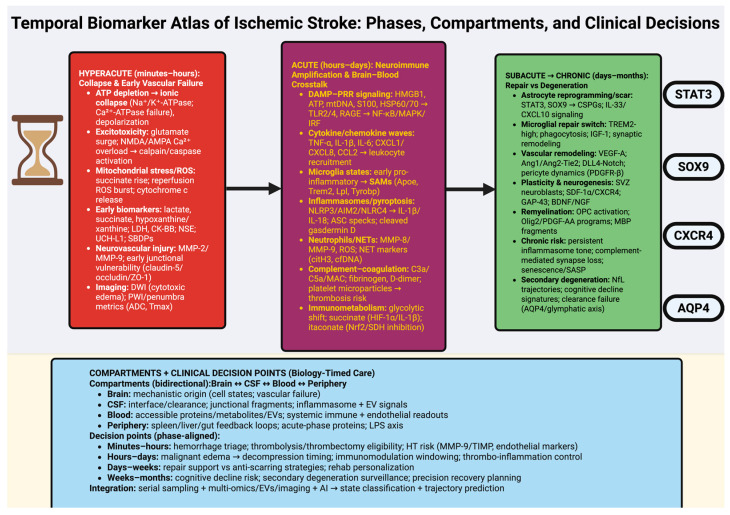
Temporal Biomarker Atlas of Ischemic Stroke. Overview of phase-specific molecular transitions, biomarker compartments (brain–CSF–blood–periphery), and corresponding clinical decision windows.

To provide a high-level overview of this framework, Figure 1 schematically summarizes the temporal phases of ischemic stroke, the key molecular transitions across each phase.

Although translation will be hampered by methodological heterogeneity, lack of standardization, and dearth of longitudinal cohort studies, temporally resolved biomarker frameworks may improve patient selection for reperfusion, optimize the timing of immunomodulation, identify neurorestorative windows [20], enable molecular phenotype stratification beyond anatomical subtype, and enhance trial design and therapeutic translation—ultimately transitioning from static reactive stroke care toward precision cerebrovascular medicine based upon the dynamic molecular logic of injury and recovery [21,22].

Therefore, this review will propose a Temporal Biomarker Atlas of Ischemic Stroke, categorizing biomarkers across the main stages of ischemic injury and recovery (hyperacute, acute, subacute, and chronic), and across biological compartments (CSF, blood, brain, and immune), treating biomarkers as dynamic, multi-compartment molecular states that can provide mechanistically timed direction in real time for interventions and facilitate the development of precision cerebrovascular medicine.

## 2. Hyperacute Phase (Minutes–Hours): Ischemic Collapse and Vascular Failure

The hyperacute phase describes the first several hours after a stroke, during which the CNS undergoes a dramatic shift from a stable homeostatic condition to an unstable condition of energy and electric instability. The initial 6 h after arterial occlusion determines, to a great extent, whether tissues will ultimately be committed to infarction (i.e., salvageable) or potentially salvaged (i.e., salvageable penumbra). Cessation of cerebral blood flow triggers very synchronous pathways of signaling among cells (neurons, glial cells, and endothelial cells) and the body, producing highly dynamic patterns of biomarkers (versus a single linear response) [23]. Therefore, it is advantageous to consider hyperacute injury as a set of overlapping molecular programs that progress simultaneously but continue to evolve with time, each with distinct patterns that provide accurate descriptions of the biological progression of the disease in real time [24].

### 2.1. Metabolic Collapse and Excitotoxic Storm: The Molecular Origin of Ischemic Injury

Collapse of metabolism is the triggering event. Oxygen/glucose deprivation inhibits oxidative phosphorylation, resulting in ATP depletion and subsequent failure of the Na+/K+-ATPase and Ca2+-ATPase, leading to the collapse of ion gradients, the influx of Na+/Ca2+, the efflux of K+, depolarization of membranes, increased levels of glutamate, impaired glutamate uptake by astrocytes, and hyperactivation of the NMDA and AMPA receptor subtypes, resulting in pathological Ca2+ loading [25,26]. Activation of calpains, phospholipases, and endonucleases by Ca2+ leads to the breakdown of the cytoskeleton and membranes, and begins a cascade of events that result in mitochondrial Ca2+ overload, and the generation of reactive oxygen species (ROS) through reverse electron transport, further decrease in mitochondrial membrane potential, and the release of pro-apoptotic mediators (e.g., cytochrome c) that lead to necrotic and apoptotic pathways [27,28]. Many of the earliest measurable biomarkers are generated by these processes: plasma/CSF lactate, succinate, and other products of anaerobic glycolysis begin to accumulate within minutes after onset and correlate with hypoxia and metabolic distress; succinate is particularly informative due to the rapid rate at which it accumulates and its mechanistic link to reperfusion-induced bursts of ROS [29]. Non-specific injury biomarkers (CK-BB and LDH) indicate the presence of membrane rupture, whereas lactate and aspartate reflect the degree of infarct and neurological deficit [30]. Ultimately, structural injury leads to the formation of spectrin-breakdown products (SBDPs), neuron-specific enolase (NSE), and fragments of cytoskeletal degradation and neuronal injury resulting from calpain cleavage. UCH-L1 peaks in hours post-infarct and is correlated with the degree of infarct and clinical outcome [31]. Although most of these candidates are non-specific to stroke, the use of temporal sampling and the selection of rationally chosen multi-marker combinations may enhance the performance of diagnostics and/or prognostics [32].

In addition to the previously mentioned candidates, other biomarkers that describe the oxidative and cell death cascades include 8-OHdG, an indicator of oxidative DNA damage; malondialdehyde (MDA), a marker of lipid peroxidation; oxidized LDL, a marker of systemic oxidative stress; and caspase-3 activity or extracellular cytochrome c, indicators of apoptosis [33]. Hypoxic compensatory signaling is characterized by HIF-1α stabilization and the initiation of protective cytoprotective programs (VEGF), and multi-omics provides additional mechanistic details: metabolomics identifies purine catabolic shifts (hypoxanthine and xanthine) that correspond to ATP breakdown and ischemic severity [34]; RNA-seq demonstrates early stress/inflammatory programs in circulating immune cells; and the expression of neuronal/astrocytic miRNAs (miR-124 and miR-9) exhibits rapid time-dependent kinetics (an early peak and subsequent decline) that relate to the expansion of the infarct—collectively describing a relatively short period of time (minutes) during which the metabolic collapse, excitotoxicity, and oxidative stress trajectories are established [35].

### 2.2. Neurovascular Unit Breakdown: Endothelial Dysfunction and Blood–Brain Barrier Failure

The disruption of the neurovascular unit (endothelium, pericytes, astrocytes, neurons, and ECM) is a hallmark of hyperacute injury. The disruption is the result of hypoxia/oxidative stress/excitotoxicity, which disrupts the tight junctions of the endothelium and initiates a self-perpetuating cycle of vascular injury that increases the vulnerability of tissues [36].

An early indicator of hyperacute pathology is the disruption of the BBB, which is the result of MMP-mediated degradation of claudin-5, occludin, and zonula occludens-1; MMP-2 and MMP-9 rapidly increase, and there is a correlation between plasma MMP-9 levels and greater infarct volume, greater rates of hemorrhagic transformation, and poorer outcomes post-thrombolysis [37]. Detection of tight-junction proteins or fragments in CSF may serve as an early indicator of disruption of the BBB. The activation of the endothelium enhances adhesion and trafficking signals, including the shedding of sICAM-1 and sVCAM-1, facilitating leukostasis and diapedesis; these markers increase early, remain elevated throughout day 1, and are correlated with permeability and inflammatory infiltration [38]. Other biomarkers of early endothelial dysfunction and prothrombotic state include vWF, thrombomodulin, and soluble E-selectin [39]. Astrocytes and pericytes exhibit rapid disruption of structure and signaling: astrocytic-end foot swelling via AQP4 contributes to cytotoxic edema, while GFAP and S100B are present in the blood within hours and serve as clinically relevant surrogate measures of astrocytic activation. GFAP has a high specificity for distinguishing ischemic from hemorrhagic stroke in the hyperacute time frame, thereby guiding acute treatment decisions [40]. Pericytes become detached from the vasculature, disrupting microvascular perfusion and worsening injury. Soluble PDGFR-beta may serve as an early indicator of pericyte pathology, while angiopoietins and ephrin ligands represent additional candidate signals of destabilization of the perivascular space [41,42].

The disruption of the BBB allows for the entry of plasma proteins, leukocytes, and peripheral cytokines into the brain parenchyma, contributing to edema and inflammation, as well as entry of CNS-derived molecules (NfL, GFAP, and neuronal exosome content) into the bloodstream as accessible surrogates of CNS injury, illustrating early bidirectional communication across the brain–blood interface [43]. Imaging biomarkers provide important additional context: PWI defines hypoperfusion/penumbra, DWI detects cytotoxic edema within minutes of onset, and derived metrics (ADC and Tmax) provide approximations of microvascular function and tissue viability. Together with molecular data, these metrics define the hyperacute state and enhance predictive capability [44].

### 2.3. Translational Biomarker Landscape: Multi-Omics Signatures, Emerging Technologies, and Future Directions

Given the inherently dynamic and time-dependent nature of hyperacute biology, clinically relevant constructs will increasingly require the integration of multi-marker, multi-omics, and multi-compartment data, rather than the evaluation of individual analytes. New paradigms that incorporate multi-omics, compartment bridging, and AI aim to capture the early programs and translate early vascular and metabolic dysregulation into actionable precision signatures in near-real time [45].

Omics studies have shown early modulation of plasma/CSF acute-phase proteins (SAA and CRP), heat-shock proteins (HSPs), annexins, and nucleic acid-binding proteins, as well as metabolomics identifying glycolytic intermediates, purine catabolites, and lipid peroxides that are consistent with the sudden metabolic arrest [46]. RNA sequencing of circulating immune cells has demonstrated early inflammatory, adhesive, and metabolic adaptation programs that are detectable as early as ~1 h poststroke, and it supports the development of composite biomarkers with enhanced diagnostic and/or prognostic capabilities [47]. Exosomes (microvesicles) represent a particularly promising source of hyperacute biomarkers, as neurons, astrocytes, endothelial cells, and platelets rapidly secrete vesicles that contain proteins, lipids, mRNAs, and miRNAs that encode cell state and injury biology (synaptophysin, GFAP, and selected miRNAs) that cross the BBB and can be detected in blood. The time-dependent evolution of cargo of exosomes provides rationale for developing phase-specific EV panels for early-stage hyperacute assessment and prognosis [48,49].

Rapid analytical platforms and computational models facilitate translation: POCT biosensors for the detection of proteins/nucleic acids in whole blood and high-dimensional machine-learning algorithms trained on multi-omics datasets can improve the discrimination of stroke subtypes, estimate infarct growth, and predict hemorrhage risk—particularly when used in conjunction with imaging and clinical data—and will develop composite signatures across multiple points of acute signal transduction [50]. Some potential applications of the technology in the next few years include using GFAP in combination with neuronal markers such as NSE to differentiate ischemic from hemorrhagic stroke before imaging [51]; selecting patients for thrombolysis/thrombectomy based on the likelihood of successful reperfusion and/or the risk of hemorrhagic transformation using biomarker-based strategies; identifying patients at risk for malignant edema early enough to allow for timely optimization of surgical decompression; and providing direction for the timing of adjunctive neuroprotective or immunomodulatory therapies based on biological state rather than on fixed time frames [52].

Challenges facing implementation include the lack of specificity of biomarkers for certain conditions and the confounding effects of comorbidities; the insufficient characterization of kinetic patterns and optimal sampling schedules; and the limited evidence base due to the small sample sizes, single-center studies, and low levels of external validation. Therefore, large longitudinal cohorts with sequential sampling, and harmonized methodologies and pipelines that combine molecular, imaging, and clinical data will be required for clinical application [6]. However, spatial transcriptomics and single-cell profiling are increasingly able to resolve cellular heterogeneity in the penumbra; EV and liquid biopsy approaches provide scalable peripheral access to central programs; and AI-driven data integration may convert early vascular and metabolic dysregulation into reproducible precision signatures that are actionable in near-real time [53]. Representative hyperacute biomarkers that correspond to molecular, cellular, vascular, and extravascular injury processes, grouped by the timing of emergence, mechanism of action, and clinical relevance, to illustrate how rapidly evolving biodynamics signals can support diagnosis, prognosis, and critical early therapeutic decision-making within minutes to hours post-sustained ischemia, are summarized in Table 1.

#### Concluding Perspective

The hyperacute phase of ischemic stroke is characterized by rapid tissue damage through hyperexcitability, leading to excitotoxicity, oxidative damage, early loss of endothelial function, and failure of cellular metabolism. Because of this rapid propagation of tissue damage, there will be a limited window of time to intervene during the hyperacute phase of ischemic stroke. Rapid onset and duration of the hyperacute phase may allow for the use of multi-modal integrated approaches, using imaging, circulating biomarkers (endothelial, inflammatory, and EVs), and clinical data to identify high-risk patients at the earliest point possible and guide neuroprotective and reperfusion therapy, as well as support individualized decision-making in the acute setting. Multi-omics profiling and AI-based models may also enhance temporal resolution of interventions in the hyperacute phase of ischemic stroke and allow for prediction of clinical outcome within the time frame that is most appropriate for stroke care [73,74].

## 3. Acute Phase (Hours–Days): Neuroimmune Activation and Brain–Blood Crosstalk

The acute phase, spanning approximately 6 h to 5 days post-occlusion, expands upon the hyperacute metabolic collapse to represent a spatiotemporal organization of the neuroimmune response through glial activation, leukocyte recruitment, endothelial reactivity, and systemic feedback mechanisms. During this time frame, the acutely ischemic brain and peripheral immune organs participate in a sustained molecular dialogue that influences both the development of secondary injury processes and the initiation of reparative remodeling. Immune responses evolve in a sequence of stages, each having different outcomes: inappropriate immune responses lead to an increased breakdown of the BBB, edema, and thrombosis; and appropriate immune responses promote the removal of debris, restoration of perilesional environments, and the initiation of tissue repair. This dynamic aspect of immune response creates a high-yielding window for the use of biomarkers for staging and identifying optimal timing for intervention [17,75,76].

### 3.1. Innate Immune Activation and Neuroinflammatory Cascades

With the onset of injury, injured neurons, astrocytes, and endothelial cells produce DAMPs (HMGB1, ATP, mtDNA, S100 proteins, and HSP60/70), activating PRRs on resident and migrating immune cells. Activation of the NF-kB-, MAPK-, and IRF-dependent transcription pathways results in the production of cytokines (TNF-α, IL-1β, and IL-6) and chemokines (CXCL1 and CCL2) that enhance local inflammation and create a chemoattractant gradient that recruits peripheral leukocytes [77]. Microglia are among the first responders to injury and have been found to express a range of transcriptional states that are intermediate between being fully active and being fully inactive [78]. Using single-cell RNA sequencing, researchers have identified stroke-associated microglia (SAMs) within ~12 h post-injury, and these microglia have been shown to have lipid metabolism and phagocytic programs (Apoe, Trem2, Lpl, and Tyrobp) that are consistent with a role in debris clearance and synaptic remodeling [79].

However, during the early stages of injury, microglia primarily produce pro-inflammatory mediators (TNF-α, IL-1β, IL-18, NO, and ROS) that contribute to injury to neurons and compromise the integrity of the BBB [80]. Activation of the inflammasome serves as a primary mechanism for the amplification of inflammation. Upon activation of NLRP3 in microglia and infiltrating macrophages, caspase-1-dependent maturation of IL-1β and IL-18 occurs, along with pyroptotic cell death, resulting in enhanced neuroinflammation. Elevated levels of IL-1β, IL-18, and gasdermin-D-containing fragments in circulation have correlated with infarct volume and clinical outcome, suggesting their potential utility as monitoring agents [81].

AIM2 and NLRC4 have also been demonstrated to be activated by DAMPs, ROS, and extracellular ATP; AIM2 specifically detects cytoplasmic double-stranded DNA released from damaged nuclei and mitochondria, thereby providing a mechanistic link between cellular injury and innate immune activation [82]. Furthermore, IL-1β, IL-18, ASC specks, and cleaved gasdermin-D can be measured in CSF and plasma, and circulating mitochondrial DNA has been demonstrated to correlate with infarct volume and clinical outcome, indicating cell-death burden as a quantifiable DAMP signature. Following the initial wave of microglial activation, microglia undergo a transition toward reparative phenotypes characterized by the production of anti-inflammatory cytokines (IL-10 and TGF-β) and neurotrophic factors (IGF-1 and BDNF) to facilitate remodeling and synaptic recovery [83]. Furthermore, the increase in soluble TREM2 in CSF and plasma ~24–48 h following injury provides an indication of microglial activation and phagocytic involvement, and changes in the content of EV cargo from microglia (miR-155 to miR-124) indicate the reprogramming of the immune response. Additionally, astrocytes also play an important role in shaping the inflammatory environment in response to injury by producing IL-33, CXCL10, and complement components that influence the recruitment of leukocytes and the activity of microglia [80,84].

Recruitment of leukocytes into the CNS represents the effector limb of this cascade. Neutrophil recruitment is mediated by CXCL1/CXCL8 and endothelial ICAM-1/VCAM-1, and neutrophils release MMP-8/MMP-9, ROS, and NETS, leading to increased BBB disruption and microvascular obstruction. NET components (citrullinated histone H3 and cell-free DNA) are used as biomarkers of thrombo-inflammation and stroke severity. Monocytes are subsequently recruited into the CNS and undergo differentiation into various macrophage states depending on the specific environmental conditions, and stroke-associated macrophages (SAMacs) have been characterized by the expression of Trem2, Spp1, and Cd163 with combined inflammatory and phagocytic properties [85]. Leukocyte recruitment is influenced by CCL2 and CX3CL1 signaling, and the CCR2+/CX3CR1+ monocyte ratio has been suggested as a predictor of infarct expansion and functional outcome. The development of these cellular trajectories are coupled to changes in immunometabolic reprogramming: microglia/macrophages switch from oxidative phosphorylation to aerobic glycolysis, and succinate (HIF-1α stabilization and IL-1β induction) and itaconate (succinate dehydrogenase inhibition and Nrf2 activation) serve as mechanistic regulators and measurable biomarkers of inflammatory phenotype [86,87].

Moreover, systemic metabolic disturbances (e.g., lactate elevation, lipid remodeling, and amino acid dysregulation) correlate with cytokine programs and clinical outcome, and integrated metabolomic–transcriptomic profiling can identify actionable immunometabolic states and therapeutic targets [88].

### 3.2. Blood–Brain Barrier Dynamics and Systemic Crosstalk

A second phase of BBB function has emerged as the post-hyperacute phase, where the BBB dynamically responds to the local and systemic inflammatory environment, and mediates repair, injury, and immune tolerance. To achieve this, all cellular components of the BBB (endothelium, pericytes, astrocytes, and ECM) must be both spatially and temporally coordinated (otherwise, there is a high risk of BBB dysfunction). In response to DAMPs/cytokine exposure, endothelial cells express adhesive molecules (selectin E, ICAM-1, and VCAM-1) that facilitate leukocyte recruitment to the site of inflammation. Plasma-soluble forms of these adhesion molecules appear in a biphasic manner (12–24 h) and therefore offer potential biomarkers of endothelial activation and subsequent leukocyte recruitment. The VEGF/HIF-1α pathway is transduced in response to inflammatory stimuli and results in increased BBB permeability and edema, and consistently elevated VEGF levels are correlated with poor outcome [89]. Angiopoietins and their binding to the Tie2 receptor continue endothelial signaling, and the downstream consequences of impaired angiopoietin signaling lead to BBB breakdown and secondary hemorrhage. Both MMP-2 and MMP-9 reach maximal levels of activity 24–48 h poststroke injury, thereby disrupting tight junctions and basement membranes, and the proteins that comprise these structures [90]. The MMP-9/TIMP ratio is a potent predictor of BBB permeability, hemorrhagic transformation, and clinical outcome. Additionally, soluble fragments of tight-junction proteins (such as claudin-5 and occludin) present in CSF and plasma serve as direct evidence of BBB disruption. The role of the meninges and CNS lymphatic system in acute stroke immunology has recently been highlighted as an important mechanism in the clearance of immune mediators and debris [91]. Meningeal macrophages and dendritic cells recognize and respond to DAMPs and cytokines in order to regulate leukocyte recruitment and antigen presentation. The removal of CSF through meningeal lymphatic vessels to deep cervical lymph nodes facilitates the removal of immune mediators and antigens, and regulates the peripheral immune response and immune tolerance. As such, novel biomarkers associated with meningeal lymphatic activity (for example, lymphatic endothelial markers such as LYVE-1 and podoplanin) represent additional candidate markers of CNS–peripheral immune communication [92].

Stroke creates a bidirectional feedback loop between the peripheral immune system and the CNS, generating factors that influence CNS inflammation. Within 12 h of stroke onset, splenic contraction begins, and the mobilization of monocytes and lymphocytes into the circulation commences [93]. Patients with normal spleen size are positively correlated with peripheral leukocyte counts, while patients with atrophic spleen may have higher peripheral leukocyte counts and worse outcomes. The acute-phase response results in the liver initiating systemic inflammation, resulting in the release of CRP, serum amyloid A, and complement proteins, which regulate endothelial behavior and leukocyte trafficking. The gut–brain axis represents yet another mechanism of interaction between the CNS and peripheral immune system. Stroke alters the motility, permeability, and microbiota composition of the gut, thereby allowing bacterial products (for example, lipopolysaccharide (LPS)) to enter the bloodstream. LPS levels in plasma within 48 h post-injury correlate with increased cytokine production, increased infarct growth, and increased mortality [94].

Complement activation reaches peak levels during the acute phase of stroke, accompanied by numerous biomarkers, including components of the C3a, C5a, and membrane-attack complex. Complement plays a critical role in the inflammatory phase of stroke by facilitating leukocyte recruitment, platelet-activated endothelial activation, and microvascular thrombosis [95]. Elevated plasma C5a concentrations prior to hemorrhagic transformation predict poor outcome. Complement-depleted mice/models show smaller infarcts. Coagulation mechanisms link inflammation and the immune response with elements of tissue-factor expression, leading to the generation of thrombin and fibrin deposition and thereby serving to amplify the immune response. Biomarkers such as D-dimer, fibrinogen, and platelet-derived microparticles reflect the prothrombotic state and predict poor outcomes [96].

### 3.3. Biomarker Landscape and Translational Implications

Acute-phase biomarker architectures include soluble proteins, nucleic acids, metabolites, EVs, imaging biomarkers, and composite multi-omics signatures that capture both inflammatory and vascular biology. Proteomics demonstrates the dynamic regulation of cytokines/chemokines, adhesion molecules, proteases, and complement factors. Panels of multiple analytes that include inflammatory, endothelial, and proteolytic markers (e.g., IL-6, IL-8, IL-10, sICAM-1, MMP-9, and complement factors such as C5a) have been shown to have good discrimination ability in small cohorts of patients with inflammatory diseases; however, the variability in the disease context and the research designs utilized limit the generalizability of findings from non-stroke inflammatory diseases (e.g., acute pancreatitis), and therefore, stroke-specific validation in larger longitudinal studies will be necessary before definitive statements regarding the prognostic utility of biomarkers can be made [97,98]. Gene-expression signatures have been associated with large vessel occlusion, risk of malignant edema, and 90-day outcomes; the combination of temporal gene expression with proteomics and metabolomics may allow for the identification of composite biomarkers with predictive power and mechanistic interpretation. Metabolomics has identified immunometabolic states (succinate, itaconate, lactate, and SCFAs) that can be used as indicators of the degree of immune activation and systemic communication [99]. Lipidomics has implicated sphingolipids and eicosanoids as regulators of inflammatory tone and vascular reactivity. EVs can be used to assess CNS and peripheral immune responses using minimally invasive techniques: neuron-derived EVs contain synaptophysin, astrocyte-derived EVs contain GFAP, and microglia-derived EVs enriched for miR-155 or miR-124 can be used to identify cellular responses [100,101]; the temporal cargo shifts in EVs may indicate changes in the transition from an inflammatory to a reparative state, and EVs may carry inflammasome-associated signals (ASC specks and gasdermin-D fragments) that are consistent with pyroptosis. Circulating microRNA panels (miR-124, miR-9, miR-155, and miR-223) are indicative of neuronal injury, glial activation, and immune polarization, and have favorable kinetics for early assessment. The accuracy of biomarkers is improved when molecular profiles are combined with imaging and functional data [102], including TSPO PET for microglial activation, complement/integrin tracers for localized inflammation, MRI-based BBB permeability mapping, and perfusion imaging for microvascular dysfunction, allowing for the creation of multimodal composites that have better predictive performance [103].

Machine-learning frameworks are likely to be increasingly applied in the translation of biomarkers to predict infarct growth, hemorrhagic risk, and recovery trajectories over time, rather than as static assessments [104]. Clinical efforts are already integrating biomarker-imaging panels for the selection of patients for reperfusion trials, for optimizing the timing of immunomodulatory therapy based on adhesion and complement profiling, and for predicting responsiveness to neuroprotective therapies using microRNA signatures [105]. While still under development, these approaches represent a feasible path toward biomarker-guided stroke care with greater predictive capabilities [106].

In conclusion, the acute phase of stroke is the period in which innate immune activation, BBB dynamics, coagulation–complement coupling, and systemic communication interact to determine if secondary injury prevails or if repair proceeds. The trajectories of biomarkers encode the mechanistic fingerprint of microglial activation, inflammasome signaling, leukocyte recruitment, endothelial dysfunction, systemic crosstalk, and immunometabolic reprogramming [107], and when integrated into composite signatures, they can guide timely therapeutic interventions. The integration of multi-omics profiling, EV and microRNA signatures, advanced imaging, and AI-assisted analysis may transform acute inflammation from an uncontrolled liability into a measurable and targetable biological state, thereby enabling the application of precision interventions guided by changing biology rather than rigid time frames [108].

## 4. Subacute Phase (Days–Weeks): Glial Remodeling and Reparative Microenvironments

The subacute phase of ischemic stroke (from approximately 5 days to 3 weeks post-onset) represents a biological turning point; it is a time of injury and repair. Acute inflammation and vascular damage begin to stabilize. Cellular reprogramming, structural remodeling, and repair signaling emerge. The subacute phase is not simply a clearance of prior damage or an acceleration of repair; it represents a true transformation where the injured CNS commences the arduous process of organizing a previously disordered environment [109]. Therefore, any activity in this phase (i.e., changes in glial phenotype, more extensive axon elimination, angiogenesis, neurogenesis, and axonal outgrowth) is of the highest priority and therefore of relevance to the development of therapeutic interventions to facilitate recovery and subsequent rehabilitation. However, like the acute phase, the emergent environment is still highly susceptible to negative environmental influences: the presence of inflammation, or chronic or widespread gliosis/scarring will interfere with the repair process and restrict functional recovery [110]. If, however, the repair and regenerative processes can be coordinated, then there is a possibility that the collective recovery will establish a basal environment conducive to long-lasting plasticity and network reorganization. Thus, measures emerging during this phase represent more than simple transient dynamic molecular signals; rather, they reflect the balance between a constellation of biological pathways, time (duration), volumetric scans, and the direction of repair signals [111].

### 4.1. Astroglial and Microglial Remodeling: Orchestrators of Tissue Repair

During the hyperacute and acute phases of stroke, the astrocytic population can become maximally functional in preparation for the beginning of the subacute phase. As the subacute phase progresses, astrocytes continue to perform additional roles in addition to releasing cytokines and maintaining the integrity of the BBB; specifically, they become involved in structural remodeling, synaptic regulation, and ECM remodeling [112]. Specifically, one of the critical components of this transition is the development of a glial scar around the infarct core 7–14 days poststroke. Originally viewed as a barrier to regeneration, glial scars are now recognized as dynamic entities that are capable of performing both protective/neuroregulatory functions (i.e., protection of necrotic tissue and inhibition of inflammatory spread) and detrimental functions (i.e., creation of a physical and biochemical block to axonal regeneration) [113].

During the subacute phase, astrocytes exhibit vast differences in regard to phenotype. Using transcriptomic analysis, astrocytes can be classified based on STAT3, Sox9, and CSPG gene expressions into various populations. Specifically, astrocytes expressing STAT3 are required for the development of glial scars and control of lesion size. Similarly, populations of astrocytes expressing CSPGs are capable of inhibiting axonal growth. The relative abundance of these populations determines the regenerative environment and can be used as a biomarker [114].

GFAP continues to serve as a useful marker of astrocyte activation, and its continued presence >1 wk poststroke indicates maladaptive gliosis. CSPGs (e.g., neurocan, versican, and brevican) are detectable in CSF and EVs, and they reflect the degree of ECM remodeling and determine axonal-regeneration potential [115]. Astrocytes also secrete substances that influence synaptic connectivity (e.g., thrombospondins (TSP-1 and TSP-2) and hevin), induce synaptogenesis, and are present in CSF during the subacute phase. Astrocytes are also responsible for maintaining metabolic and ionic homeostasis by regulating glutamate transport via EAAT1/2 and potassium balance via Kir4.1 [116]. Microglia, the resident immune cells of the CNS that mediate the initial pro-inflammatory response to ischemic injury, undergo radical changes in morphology during this phase of subacute recovery. The pro-inflammatory state of the acute phase gives way to “repair” states characterized by the upregulation of genes involved in phagocytosis, lipid metabolism, and tissue remodeling [117]. Failure to clear dead neurons, myelin, and synaptic debris precludes the establishment of a repair context. MerTK and Gas6, molecules involved in recognition of apoptotic cells and phagocytosis, are significantly increased in this phase and are detectable in CSF. Additionally, TREM2, a non-specific indicator of microglial reactivity and phagocytosis, reaches its peak in soluble form between 7 and 14 days poststroke and correlates with functional recovery. Although microglia function primarily in cleaning, they contribute to the reorganization of synaptic networks by promoting the pruning of synapses, labeling synapses destined for elimination with complement proteins C1q and C3, and facilitating the reorganization of functional connectivity [118]. The concomitant elevation of C3a in CSF, along with the indication of continuous pruning of downstream circuits, suggests that subacute microglia are continuously eliminating redundant synaptic connections. Microglia-derived EVs labeled with synaptic or complement markers may represent XM candidate biomarkers for this phenomenon. Finally, microglia have been implicated in the provision of trophic factors (e.g., IGF-1 and BDNF) to neural progenitors, thereby influencing their maturation into functional neurons or oligodendrocytes; these factors appear in CSF and plasma, and they suggest that the microglial-derived signals may indicate reparative activity within the brain, although the specific types of reparative activity (i.e., neurogenic vs. remyelinating) cannot be determined. Astrocytes and microglia are the primary contributors to the subacute phase of recovery. During this phase, astrocytes (through IL-33 and TGF-β) and microglia (through IL-10 and growth factors) engage in signaling patterns that direct one another’s behavior and regulate the composition of the extracellular matrix. Changes to this signaling pattern may favor deleterious responses, such as sustained IL-1β or TNF-α signaling that promotes the development, continuation, and/or maladaptive glial scarring. Conversely, signaling patterns of IL-33, TGF-β, and soluble cytokines are indicative of the type and direction of the repair [119].

During the same time frame as the emergence of reparative signals, the subacute phase is characterized by the activation/initiation of reparative programs aimed at restoring perfusion, replenishing lost cells, and repairing neural networks. Angiogenesis, neurogenesis, oligodendrogenesis, and axonal plasticity occur simultaneously and are regulated by inflammatory and glial signaling [120].

Revascularization is a significant component of the subacute phase. The combination of hypoxia and inflammation stimulate angiogenic factors, such as vascular endothelial growth factor (VEGF), angiopoietins (Ang1 and Ang2), fibroblast growth factor-2 (FGF-2), and stromal-derived factor-1α (SDF-1α), to drive endothelial proliferation, migration, and tube formation in order to bring new capillaries into neovascular networks to connect to the original vasculature. VEGF increases in plasma and CSF starting at approximately day 3 poststroke and correlates with new vessel formation and repair [121]. Overexpression of VEGF may disrupt the integrity of the endothelial layer to some extent, and the timing and concentration of VEGF may be important. The maturation of vessels is also regulated by angiopoietin signaling. For example, Ang1 stabilizes vessels, while Ang2 allows for their remodeling. The ratio of Ang1/Ang2 in peripheral blood may serve as a longitudinal biomarker for vessel development [122].

### 4.2. Angiogenesis, Neurogenesis, and Network Reorganization

Matrix metalloprotease (MMP)-2 and MMP-9 are consistently expressed in periinfarct tissues. The degradation of the basement membrane, allowing for endothelial migration, is achieved by the action of MMP-2 and MMP-9. The ratios of MMP-2/MMP-9 and TIMP-2/TIMP-1 indicate a balance between remodeling states and stable states. Endothelial progenitor cells (EPCs) are mobilized from the bone marrow by SDF-1α and G-CSF and are found in greater abundance poststroke, peaking in the subacute phase and localizing to brain tissue [123]. EPCs, namely CD34 and VEGFR2, may serve as indicators of angiogenic activity. Pericytes play a role in the stabilization of capillaries and the function of the BBB. Soluble PDGFR-β may indicate pericyte levels of activity. Pro-angiogenic mRNAs and microRNAs (miR-126 and miR-210) in endothelial EVs reflect the remodeling characteristics of angiogenesis [40].

Although much of the neuronal loss is irreversible, the adult brain retains limited potential for some forms of neurogenesis. The primary sites of neurogenesis in the adult brain are located in the subventricular zones and subgranular zones. Following ischemic injury, neural progenitors are activated and proliferate, migrating to peri-infarct areas, producing new mature neurons and glia. Peaks in neurogenesis occur between 7 and 14 days poststroke and are driven by ectopic neurogenic-promoting factors, such as cytokines, growth factors, and ECM states and signals, which possess pro-neurogenic propensity [124]. Like EPCs, markers of neurogenesis, such as DCX, PSA-NCAM, and nestin, are found in higher levels in peri-infarct brain and are detectable in CSF. BDNF and VEGF elicit both the division and migration of neural progenitors; accordingly, the plasma or CSF levels of either correlate with spontaneous neurogenic activity. SDF-1α/CXCR4 signaling directs the migration of neuroblasts to the SVZ, and plasma SDF-1α may correlate with neurogenic activity [125]. Furthermore, the regulatory effects of microglial and astrocytic activities may be relevant: microglial-derived IGF-1 and TGF-β may enhance the survival of neuroblasts, whereas CSPGs produced by astrocytes may inhibit the migration/infiltration of neuroblasts. Biomarker profiles containing trophic factors, ECM components, and chemokines provide a temporal view of the potential for neurogenesis [126].

Functional recovery requires not only new neurons but also reorganization of surviving networks. Network reorganization involves axonal sprouting and synaptic reorganization in the peri-infarct cortex, and surrounding cortical regions, as new pathways, are generated to bypass damaged circuits. For example, axonal growth can be monitored using GAP-43, a marker of growth that is elevated between days 5 and 7 poststroke and maintained for several weeks. Elevated GAP-43 in CSF correlates with functional recovery [127]. Axonal growth is influenced by an interplay of pro-growth signals and inhibitory signals. Pro-growth signals include neurotrophins (BDNF and NGF), which promote axonal extension, whereas myelin-associated inhibitors (Nogo-A, MAG, and OMgp) and their receptors inhibit growth. Soluble Nogo receptor fragments and myelin debris factors present in the CSF may reflect potential for regeneration. Activity of matrix MMPs, reflected by MMP/TIMP ratios, facilitates penetration of axons through ECM barriers [128].

**Figure 2:**
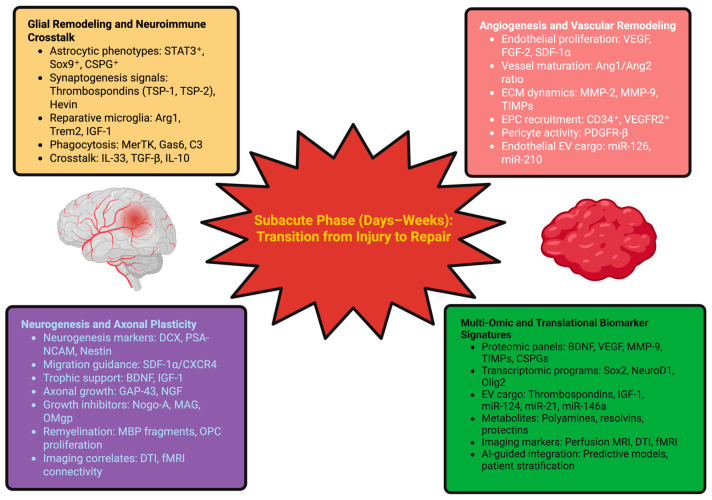
Subacute phase of ischemic stroke (days–weeks): Transition from injury to repair. A schematic overview of the major biological programs and biomarker signatures that define the subacute phase. Astrocytic and microglial remodeling orchestrate the transition from inflammation to repair through cytokine signaling, synaptic modulation, and extracellular matrix restructuring. In parallel, angiogenesis restores perfusion; neurogenesis and axonal plasticity rebuild neural circuits; and multi-omics biomarker signatures capture the evolving reparative landscape. Integration of these processes provides opportunities for prognostication, patient stratification, and phase-specific therapeutic interventions.

OPC proliferation and differentiation occurs during this time and are the basis of myelination/remyelination of axons. Myelin basic protein (MBP) fragments can be found in the CSF as a measure of myelin turnover, while oligodendroglial-derived EVs containing myelin proteins and regulatory RNAs provide a minimally invasive way to measure dynamics associated with remyelination [129]. Network reorganization occurs beyond the injury boundary: remote areas undergo structural and functional changes, a process is termed diaschisis. Imaging functional connectivity reveals changes in network connectivity over time, while biomarkers like synapsin, PSD-95, and Arc are associated with synaptic plasticity. Monitoring these processes with biomarker panels may give insight into recovery potential early and guide rehabilitative treatment [130]. To exemplify and provide not only the complexity but the interactivity of these mechanisms, Figure 2 attempts to present a schematic overview of the subacute phase, which gives prominence to the key cells, molecular pathways, and representational biomarker signatures that shape the human repair map and render insights for prognosis, patient stratification, and therapeutic timing.

A large number of proteins, nucleic acids, metabolites, EVs, imaging parameters, and multi-omics integrative signatures define the biomarker landscape of the subacute phase of stroke, which defines the biology of repair, while providing a basis for prognosis, patient stratification, and therapeutic monitoring [131].

Proteomic studies have shown significant changes in a variety of proteins, including growth factors, cytokines, components of the ECM, synaptic proteins, etc. Panels of biomarkers such as BDNF, VEGF, Ang1/2, MMP-9, TIMPs, and CSPGs suggest that ECM remodeling, angiogenesis, and scar formation are occurring in the injured brain. Timing is important, as MMPs and CSPGs are increased early in the post-injury period, suggesting an increase in the likelihood of scar formation, whereas late increases in neurotrophic factors and angiogenic markers suggest recovery of the damaged area [132]. When using transcriptomics, concurrent transcriptional profiles include those representing gene-expression programs of neurogenesis, axon growth, and glial remodeling. Upregulation of Sox2, NeuroD1, and Olig2 indicates activation of the neurogenic and oligodendrogenic gene-expression programs. Composite biomarkers can be developed when using both transcriptomics and proteomics to define composite biomarkers with both improved predictive power and mechanistic understanding [133]. EVs are also intercellular messengers—and can be used as biomarkers—in intercellular communication. Astrocytic EVs enriched for synaptogenic proteins (e.g., thrombospondins) indicate synaptic remodeling, whereas endothelial EVs enriched with angiogenic miRNAs (miR-126 and miR-210) provide evidence of neovascularization. Microglial EVs containing IGF-1 and anti-inflammatory miRNAs indicate that the microglia are transitioning toward a repair phenotype. Circulating microRNAs (e.g., miR-124 (neurogenesis), miR-210 (angiogenesis), miR-21, and miR-146a (resolution of astrocyte-mediated inflammation)) are dynamically increased and can be used as a panel of composite microRNAs to track repair and predict clinical recovery [134]. Metabolic reprogramming underlies many of the repair mechanisms. Metabolic profiling has demonstrated increased levels of polyamines, sphingolipids, and amino acids involved in proliferation and differentiation. Increased levels of lipid mediators, such as resolvins and protectins, derived from omega-3 fatty acids occur during the subacute phase to mediate the resolution of inflammation and promote repair. The plasma levels of these mediators may reflect the transition from a destructive inflammatory response to a regenerative response [135].

Imaging provides additional spatial and functional information. Perfusion imaging can assess neovascularization. Diffusion tensor imaging (DTI) and tractography can assess white-matter integrity. Functional magnetic resonance imaging (fMRI) can evaluate network connectivity. The use of these imaging modalities in combination with molecular data will enhance predictive accuracy and further elucidate the complex nature of repair. The ultimate goal of subacute biomarker research is the creation of composite signatures incorporating multi-omics, imaging, and clinical data [136]. Machine-learning algorithms will utilize longitudinal datasets to recognize patterns that are predictive of outcome, stratify patients according to their capacity for regeneration, and remove the uncertainty regarding therapeutic timing. A model combining BDNF, VEGF, GFAP, and EV dynamics with imaging biomarkers of connectivity will enhance predictive capabilities compared to traditional scores derived from these laboratory-based measures to predict outcome at three months [137]. Utilizing biomarker signatures to optimize study design will revolutionize how individuals are selected for neurorestorative therapy with timely intervention. Biomarkers of maladaptive gliosis will allow for the identification of those who might benefit from anti-scarring treatments. Injury angiogenic and neurogenic signatures will provide the signatures necessary to direct the delivery/mobilization of trophic factors or progenitor cells [138].

In essence, the subacute phase of stroke is a transformational period where the brain is attempting to renovate itself at a molecular and network level. This process includes glial reprogramming, angiogenesis, neurogenesis, and axonal remodeling, each of which holds promise for recovery and contains the seed of potential maladaptive repair. Therefore, the emerging biological-based biomarkers and bio-signatures developed by dissecting this multi-layered chart represent more than just an existing representation of biology; rather, they capture the very essence of repair—the timing, direction, and efficacy of repair responses [139]. Accurate interpretation of this coding system represents a paradigm-bending opportunity, enabling clinicians to view the brain’s reparative trajectory, identify those who may benefit from targeted treatments, and develop targeted therapies that evolve to meet the changing biology of (recovery). As the multi-omics era continues to converge with spatial transcriptomics and advanced imaging, the subacute phase of stroke will eventually cease to be a “black box” of ambiguous recovery and will become a precisely mapped terrain for treatment interventions. Ultimately, deciphering the biomarker-based signatures of the subacute phase will be key to translating stroke rehabilitation from a catastrophic event into one that can not only be treated acutely, but dynamically—in sync with the brain’s repair attempts [140].

## 5. Chronic Phase (Weeks–Months): Plasticity, Maladaptation, and Long-Term Biomarker Signatures

Although the chronic phase of ischemic stroke (weeks–months) is often viewed simply as the delayed sequelae of the prodrome, it represents a distinct biological Act II. In fact, the brain is actively engaged during this time in the process of both regenerative plasticity (e.g., volumetric recovery) and the degenerative phase, and with structural reassembly (e.g., restitutive axonal integrity) and chronic inflammation, as well as latent resilience and accelerating demise [141]. At this point, the nervous system is working to restore the brain’s architecture, retrain the brain’s circuitry, and recover function. Although the connections formed allow for recovery, the pathology associated with the disconnections depends on the degree of misalignment [142]. Therefore, the biomarkers generated during this period are not static and phantom indicators of past disruption; rather, they represent dynamic signaling regarding the current trajectory of poststroke recovery, including the rate, direction, and quality of poststroke recovery [143].

### 5.1. Network-Level Plasticity and Epigenetic Remodeling

During the chronic phase of recovery, the brain’s ability to undergo widespread reorganization is the main focus of interest. The surviving neurons continue to exhibit chronic axonal sprouting and synaptic remodeling; the endogenous survival of neurons results in the increase in the number of neurons, as well as the creation of a new connective map for the lost pathways. The chronic structural plasticity indicator is represented by the elevated levels of low-MW and low/high-MW proteins (e.g., GAP-43, SPRR1A, and βIII-tubulin), for example, for many weeks/months following the acute-phase time points [144]. The intermediate neurotrophins (e.g., BDNF and NGF) mediate spine turnover and new synaptic stability, and the plasma and CSF levels of intermediate neurotrophins are correlated with functional outcomes. Transcortical reorganization exceeds the peri-infarct cortex: new transcallosal connective fibers in the zone of deafferented areas; new corticothalamic, corticostriatal projections synapse following sprouting; and the previously expressed network hub genes are redirected [145].

This basic reorganization is further elaborated through activity-dependent molecular metrics. The activation of CREB, CaMKII, and NMDA receptor subunits elicits long-term potentiation-like changes in medial olfactory gyrus neurons, and the elevation of presynaptic scaffolding proteins (PSD-95 and Homer) elevates EV cargo to serve as a minimally invasive index of synapse potentiation. Functional imaging can also include index, in which resting-state fMRI identifies the formation of nascent network connectivity, and DTI demonstrates that there are fractional increases in anisotropy along restructured hit tracts [146]. White-matter plasticity may be equally important. Directed oligodendrocyte precursor cells proliferate and differentiate via signals of Olig2, Sox10, and PDGF-AA to entrap new axon sprouts in myelination in order to optimize action potential conduction. The CSF concentrations (or inadvertently from EV sources) of myelin oligodendrocyte glycoprotein and oligodendrocyte-derived EVs are notable evidence of the remyelination of axons. Decreases in microstructural integrity, recorded as an emergence of decreased radial diffusivity, occur rapidly prior to function [147].

In addition to structural and, possibly, microstructural changes, stroke may create a life-altering epigenetic memory of the injury, which may limit the deficit potential of plasticity. Some epigenetic “marks” include changes in DNA methylation in the promoters of Bdnf and Gap43; a change in chromatin post-translational modification, such as H3K27 acetylation; and the active modification by lncRNAs and circRNA in order to reprogram transcriptional responsiveness. These epigenetic marks on cell-free DNA, environment, and circulating RNA measures may be biomarkers of latent neuroplastic potential and may be utilized to determine which populations are most predisposed to delaying rehabilitation or epigenetic manipulation therapy [148].

### 5.2. Maladaptive Remodeling, Secondary Degeneration, and Cognitive Decline

There is a shadow aspect to plasticity’s restorative potential; ultimately, the same mechanisms drive pathology if unimpeded. Astrocytes may continue to express GFAP and vimentin and reside in producing CSPGs (neurocan and brevican), which develop an inhibitory matrix to superficially block axonal regeneration. Microglia may continue to be chronically activated, as evidenced by observable, longitudinally sustained IL-1β, TNF-α, and ROS behavior, along with failure in phagocytosis [149]. Soluble TREM2, sCD14, and TSPO-PET activity indicate that this maladaptive state continues. Active complement activation occurs, and both C1q- and C3-dependent synaptic pruning destabilize the network. Chronic inflammation is maintained in a cyclic “feed-forward” status of damage [150]. Sustained NLRP3 activation and DAMP release culminate in continuously pro-inflammatory glial cells, which are markers of broadband markers of senescence; p16^INK4a; p21; and SASP (IL-6, GDF15, and HMGB1). Senescence-associated profiling of periphery and EVs, to match with cognitive decline, suggests that the peripheral associations are predictive. Cytokine and immune dysregulation occur in the periphery: Th17 expansion, Tregs contraction, and IL-17A higher + sIL-2R are considered systemic components of neuroinflammation, yet neuroinflammation is ongoing [151]. 

Secondary neurodegeneration expands the extent of the damage related to stroke and is now beyond the original lesion site. Wallerian degeneration, trans-synaptic spread of pathology, and chronic inflammation result in progressive secondary injury to other areas of the brain, specifically the hippocampus and thalamus. Plasma and CSF NfL are elevated for months; inflammation and microglial activation appear to spread and contribute to repair failure, and evidence of myelin-breakdown products indicates repair failure; and MRI exhibits thalamic atrophy, hippocampal atrophy, and tract-specific degradation in microstructural integrity, connecting remote degeneration and overall loss of function [152]. Cognitive decline appears to be caused by a combination of all of the above processes. Biomarkers linked to neurogenerative diseases—phosphorylated tau, Aβ42/40 ratio status, and neurogranin—are worse in poststroke patients and their cognitive outcomes. These biomarkers, and volumetric and connectome correlates will, potentially, eventually identify patients who are at a very high risk for poststroke dementia. Neurodegenerative mechanisms also present several opportunities for intervention prior to the occurrence of irreversible cognitive decline [153].

### 5.3. Long-Term Biomarker Signatures and Precision Therapeutic Windows

Processes in biology are currently occurring over weeks and months, and therefore, longitudinal biomarker monitoring is particularly useful. The continued elevated levels of BDNF, VEGF, and GAP-43 illustrate continued neuroplasticity and positive outcomes, whereas continued elevated levels of CSPGs, MMP-9, and inflammatory cytokines illustrate maladaptive remodeling. Multimodal composite panels (proteomics, transcriptomics, metabolomics, and epigenomics) far exceed the utility of traditional clinical measures and reveal transitional triggers between regenerative and degenerative trajectories [154]. An area of rapid development is the identification of “biological clocks,” based on epigenetic marks, EV cargo, and transcriptomic shifts. Temporal biomarkers quantify the biological age of the poststroke brain and the optimal therapeutic window. Highly increased neurogenic microRNAs may identify the optimal timing for rehabilitation, whereas CSPG-EV-associated peaks may guide anti-scarring interventions [155].

Machine learning applied to longitudinal data is identifying the underlying biological states—plasticity-predominant, inflammation-persistent, degeneration-vulnerable, and mixed remodeling—that are capable of stratifying patients with greater specificity than ever before. These states may also provide avenues of treatment; for example, inflammatory persistent profiles may be amenable to late immunomodulation, whereas plasticity-predominant states will likely benefit the most from intensive neurorehabilitation therapies or neuromodulatory therapies [156]. Predictive algorithms combining molecular, imaging, and digital data have the capability to move therapy timing and intensity forward with greater precision than traditional clinical time frames [157].

The greatest promise of chronic-phase biomarkers is to move care from reactive therapy toward adaptive care. For example, a high IL-1β or NLRP3 could indicate that inflammasome therapy should be initiated; CSPG or TGF-β profiles may direct you to use anti-scarring options; and aberrantly high or increasing BDNF or VEGF may direct you to use additional neuromodulation or growth factor therapy. The digital biomarkers embedded in wearable devices and smart phone/cognitive/speech assessments add continuous functional context, enabling patient care to be adapted to the individual patient’s biological state [158]. Table 2 is proposed to provide a means of organizing biomarkers and mechanisms that characterize the chronic phase of ischemic stroke in a more organized manner to facilitate a better understanding of the complex and evolving processes that are involved. Table 2 highlights how the regenerative and maladaptive trajectories evolve over time, from synaptic remodeling and myelin regeneration to chronic inflammation, glial senescence, and secondary degeneration, and how those events can be translated into measurable molecular and imaging biomarkers.

#### Perspective

The chronic phase is now an integral part of the stroke biology process and no longer just a final stage; it is an ongoing chapter that updates the balance of regeneration and degeneration in the long term. Biomarkers that are used over the long term provide more than a measure of the ongoing balance between regeneration and degeneration; they also provide a narrative of the development of epigenetic memory within neural networks and the reconsolidation of structural components of the brain, of the subsequent senescence and plasticity of synapses, and of the eventual remodeling of neural networks back to their original cognitive tone. The convergence of multi-omics combinations, connectome investigations, and machine-learning technologies with the proprioceptive neural circuitry that controls stroke and cognitive physicality poststroke will replace stroke as a short-lived emergent condition with a long-lived chronic condition of measurable and editable phenomenon. This recovery should be less about relying on the phenotypes of the brain and more about utilizing the biology of the brain itself [172].

## 6. Biomarkers Across Biological Compartments: From Brain Tissue to Blood and CSF

Stroke biology does not take place within the confines of one anatomical location; instead, it is a spatially dynamic process that commences from the damaged brain parenchyma, continues via the interstitial space and CSF, and finally reaches systemic circulation. The various biological compartments (from the environment surrounding the peri-infarct cortex to the perivascular glymphatic channels and eventually blood) enable a distinct and/or supplementary perspective of the evolving pathophysiology [173]. Together, they form a continuum of molecular stories about the disease progression, from acute vascular injury to chronic disease state. The biomarkers in each compartment exhibit differences regarding accessibility, temporal characteristics, mechanistic relevance, and translation, and together they collectively deliver a full picture of the disease, thus requiring biological knowledge to achieve timely intervention [174].

### 6.1. Central Biomarkers: Brain Tissue and Interstitial Fluid

The site of stroke pathobiology is in the infarct core and peri-infarct region. The molecular signature in this area develops immediately after ischemia due to oxidative stress, ionic failure, and proteolytic cleavage, which produce a constellation of molecules, including damage-associated molecular patterns (DAMPs), excitatory neurotransmitter-related signaling molecules, and stress-inducible transcriptional regulation of gene networks. The peri-infarct region generates an inflammatory response; synaptic reorganization; angiogenesis; and, ultimately, neurogenesis, which determines the fate of the tissue [175].

Recent advances in single-cell multi-omics and spatial transcriptomics have provided new insights into the intricate spatiotemporal complexities of injury. Microglia undergo a shift from a pro-inflammatory activation profile, which involves the genes Nlrp3, Il1b, and Ccl2, to a reparative microglial subset that involves the genes Trem2, APOE, and Spp1. Activated astrocytes increase protein production of Stat3, Sox9, and Serpina3n, but they switch from activated astrocytes to scar-forming astrocytes [176]. Endothelial cells are involved in the induction of angiogenic pathways (Vegfa and Dll4), while neurons induce growth-enabling pathways (Atf3, Sox11, and Gap43), all of which can undergo post-translational modification (tau phosphorylation, H3K27 acetylation, and SUMOylation of synaptic proteins), providing specificity to biomarkers and establishing connections between molecular signatures and biological processes [177].

In addition to the previously described individual biomarkers, spatially resolved interaction maps have detailed the ligand–receptor networks responsible for multicellular responses. For example, microglia–endothelial interactions (CSF1-CSF1R and VEGFA-VEGFR2), astrocyte–neuron interactions (THBS1-α2δ1 and TGF-β-TGF-βR2), and oligodendrocyte–microglia interactions (IL-33-ST2) improve angiogenesis, synaptogenesis, and myelination. These molecular signals could be considered the basic units of higher-order biomarker modules that would add more value to biomarkers than simply being individual molecules [178].

The interstitial fluid (ISF) that surrounds brain cells provides a dynamic reflection of these events in real time. Studies using microdialysis have shown the presence of peak levels of glutamate and aspartate during the early phase of ischemia, followed by a lactate-to-pyruvate transition; a release of multiple DAMPs (such as HMGB1 and S100B); and, finally, inflammation-related biomarkers (such as cytokines, e.g., IL-1β and TNF-α; and proteases, e.g., MMP-9) and restorative biomarkers (such as BDNF and VEGF) [179]. Additionally, ISF can transport EVs that contain microRNAs and regulatory proteins, and they can capture information from cellular states that determine clinical endpoints prior to becoming evident in imaging techniques. Recent microfluidic technologies now provide the possibility to continuously monitor ISF biomarkers in real time and at the bedside during physical therapy [180].

### 6.2. Cerebrospinal Fluid: The Molecular Interface of Brain and Periphery

CSF occupies a functional position that enables it to interface with both central and peripheral biology. Due to its proximity to the CNS and repeated sampling opportunities, CSF is exposed to CNS-type signals in real time. During the stroke time course, the proteome of CSF is dynamic. Early increases in GFAP, S100B, UCH-L1, and NSE are indicative of early astrocytic and neuronal injury, respectively, while MMP-9, Claudin-5 (between endothelium), and thrombin–antithrombin complexes are indicative of BBB dysfunction and breakdown of the NVU [181]. At later time points, VEGF, FGF-2, and Ang-1 are indicative of angiogenesis, while BDNF, GDNF, and IGF-1 are indicative of neurogenesis and synaptic plasticity. Increased CSPG and complement factors (C3a and C5a) are indicative of astrogliosis and active synaptic pruning. Assays measuring proteoforms, i.e., phospho-tau vs. total tau, and active vs. latent MMP-9, will provide further mechanistic insight [182].

EVs present in CSF also provide added complexity. Neuronal EVs contain synaptic-associated proteins and microRNAs (e.g., miR-132 and miR-124), indicating plasticity. Astrocytic EVs retain CSPGs and thrombospondins, and microglial EVs have miR-155- and inflammasome-relevant components that indicate pyroptosis. Recently, the ability to separate individual vesicles has allowed for the identification of cell type-specific EV subpopulations and the formulation of composite EV panels that cover the breadth of inflammation versus repair. Non-coding RNA and epigenetic cargo are expanding the biomarker portfolio. Panels that include miR-124, miR-9, miR-210, and miR-21 indicate neurogenesis, hypoxic response capacity, and glial reactivity [183]. Long noncoding RNAs such as MALAT1 and MEG3 regulate angiogenesis and apoptosis, while circular RNAs (circHIPK3 and circDLGAP4) regulate endothelial stability and synapse maintenance. Lastly, epigenetic fragments (methylated DNA and histone) collide with EVs, illustrating the transcriptional history (memory) and potential plasticity. The glymphatic pathway that regulates CSF-ISF balance and macromolecular clearance is involved in the CSF-biomarker continuum [184]. Disrupted clearance of proteins and metabolites may relate to stroke-induced disruption. AQP4 isoform ratios, perivascular tracer-signaling kinetics, and clearance of exogenous tracers may serve as biomarkers of glymphatic function. Metabolomic profiling demonstrates changes in succinate and itaconate (immunometabolism), kynurenine and tryptophan (microglial activation), and SCFAs (gut–brain signaling). Ultimately, we cannot confidently state that not all metabolites are innocent victims of inflammation and repair, as some may serve as potential therapeutic targets [185].

### 6.3. Peripheral Biomarkers: Translating Central Pathology into Accessible Signals

Peripheral blood biomarkers, with the most clinically practical application, translate central events generated by the CNS to accessible and diverse forms of analytical data. Since peripheral blood contains baseline systemic noise, along with whatever factors in that systemic noise in this biofluid that correlate with the significance of central pathology, it is connected to injury, inflammation, and repair. Elevated GFAP, UCH-L1, and NSE levels during the early phases of stroke represent the rapid response signals for astrocytic and neuronal injury [186]. Elevated levels of MMP-9, ICAM-1, and VCAM-1 during the early phases of stroke represent BBB dysfunction and leukocyte-infiltration events. S100B persists as an indicator of astrocytic activation, and elevated BDNF, VEGF, and IGF-1 represent repair. Increased levels of CSPG indicate glial scarring [187]. Elevated C5a levels may represent hemorrhagic vulnerability. IL-6, TNF-α, and IL-1β levels during chronic neuroinflammation or cognitive impairment represent chronic neuroinflammation or cognitive impairment. By integrating biomarker trajectories (reducing injury-related factor(s) and increasing plasticity factor(s)), clinicians can provide a better prognosis for clinical outcomes [188].

Peripherally derived blood-based immunity consistently modulates CNS-based immunology. Neutrophil-to-monocyte-to-T-cell activation, Th17 T-cell expansion, contraction of Tregs, and exhausted CD8+ T cells represent chronic inflammation and systemic immunosuppression. Single-cell transcriptomics of peripheral blood mononuclear leukocytes can identify the programs regulated by hypoxia (Hif1a and Slc2a1), cytokines (Il1b and Ccl2), and exhaustion (Pdcd1 and Lag3) that correlate with peripheral immune tone. Circulating LPS, SCFAs, and gut metabolites are indicators of gut dysbiosis and CNS inflammation, and initiation of recovery trajectories. Circulating EVs disseminate CNS-derived factors to peripheral tissues [189]. Neuron-derived EVs contain synaptic proteins and miR-132 or miR-124; astrocytic EVs contain GFAP or CSPG; and microglial EVs contain inflammasome dynamics, reflecting their role in repairing injuries. Non-viable nucleic acids (cfDNA, cf-dans mtDNA, and microRNA) are released, where degrees of neuronal necrosis are represented by cf-mtDNA; and miR-21, miR-155, and miR-210 represent angiogenesis, increased inflammation, and metabolic reprogramming. cfDNA DNA methylation signifies long-term transcriptional events and epigenetic memory [190].

**Figure 3:**
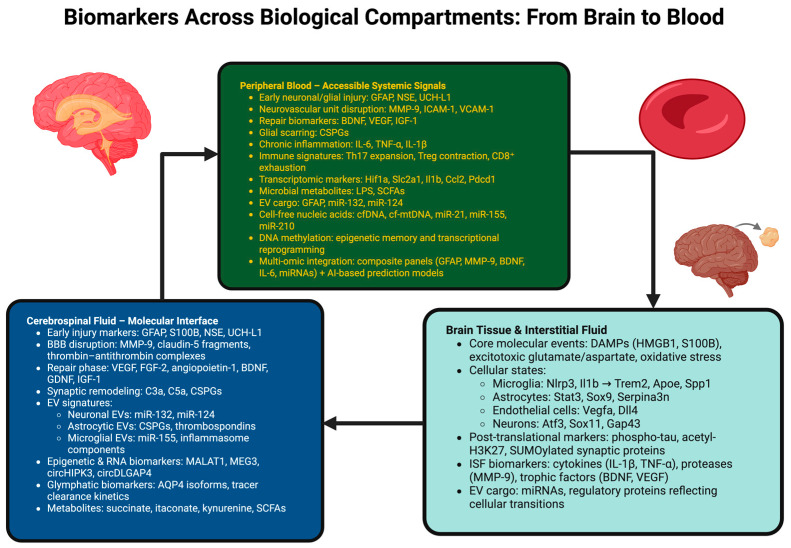
Biomarker propagation across biological compartments following ischemic stroke. Molecular signals originate in the injured brain parenchyma and interstitial fluid (ISF), where cellular states, ligand–receptor interactions, and post-translational modifications shape the evolving pathophysiology. These signals are relayed to the CSF, a privileged molecular interface capturing injury markers, extracellular vesicle cargo, noncoding RNAs, and metabolites that reflect neuroinflammation, angiogenesis, and plasticity. Peripheral blood provides an accessible clinical window, translating central events into systemic biomarkers, including cytokines, cell-free nucleic acids, immune signatures, and microbial metabolites. Integrated across compartments, these markers offer a dynamic multi-omics view of stroke evolution and enable predictive, biology-guided interventions.

Multi-omics has the potential to significantly improve the predictive capability of peripheral biomarkers. A composite (GFAP, MMP-9, BDNF, IL-6, and microRNA) panel predicts infarct size, cognitive decline, and recovery of function with high accuracy [191]. This progression of biomarker propagation across biological compartments following ischemic stroke is captured in Figure 3, which conveys how these biomarkers traverse the brain–CSF–blood axis and summarizes the preliminary stroke biology and predictive, multi-omics diagnostic nexus.

#### Reflections and Outlook

Biomarker migration from the brain to peripheral regions is not just a diffusion of molecular signals; it is a biological story that unfolds. Brain-derived signals explain the mechanistic nature of stroke, but they are difficult to find. Biomarkers derived from CSF at the brain–body interface provide the highest fidelity for describing the neuroinflammatory and neurogenetic/neuroplastic aspects of the biology. While peripheral biomarkers are more distal in time, they connect the molecular impacts of stroke to the clinically relevant and actionable outcomes [186]. The future will include combining these levels and using a systems approach to integrate the molecular biology of cascades in the brain, and the proteomic surfaces of CSF, glymphatic flow, and immune responses in the blood. Ultimately, the integrated biomarkers will go beyond the linear representation of the event, connecting therapy to a continuum of measurable states—referred to as the “pathobiome”—and the biomarker paradigm will allow us to measure, predict, and ultimately manipulate the pathobiome continuum. This process of stroke and all other biomarker proxies will help us to describe the continuum of biomarker proxies that represent anticipated treatments [192].

## 7. Emerging Technologies and AI-Driven Biomarker Discovery

Biomarker discovery has evolved significantly over the past ten years—from a relatively limited protein-focused discipline to a highly technological, high-volume precision medicine field at the intersection of high-resolution biology and computational intelligence. Stroke is an exemplary model system for testing these emerging methodologies. Today, we are witnessing an explosion of biological measurement capabilities that range from single-cell to whole-organism levels, coupled with artificial intelligence systems designed to extract meaningful information from large numbers of diverse data [193]. Ultimately, this convergence of technologies is poised to bring about a new era of cerebrovascular medicine, where complex molecular processes become clinically actionable and biological knowledge is translated into applied precision care [194].

### 7.1. Frontier Technologies for Biomarker Discovery

By identifying cell type-specific injury and repair mechanisms via single-cell and single-nuclei multi-omics, poststroke biomarker discovery has dramatically advanced. Single-cell RNA sequencing in the peri-infarct cortex shows sequential activation of microglial states, for example, an early inflammatory phase characterized by a pro-inflammatory program (e.g., NLRP3, IL1B, and CCL2), followed by repair and removal phases (e.g., TREM2-high and APOE-positive) [195]. In addition, there was also substantial heterogeneity observed within each of the other major glial cell types (astrocytes, neurons, and oligodendrocytes) [196]. Importantly, these cellular states are reflected in the CSF and EV cargo, thereby allowing for non-invasive sampling of changing brain states [197].

By combining single-cell RNA sequencing data with chromatin accessibility and histone modification data, researchers are able to begin to identify the regulatory logic driving these changes in cellular states, thereby allowing for a more informed interpretation of candidate biomarkers and for the ability to temporally stratify based on mechanisms [198]. Additionally, spatial multi-omics provides anatomical context to map discrete microenvironments, such as the astroglial barrier zone, VEGFA/DLL4 angiogenic niches, and TREM2/SPP1 phagocytic regions [199]. These provide a way to link circulating biomarker signals to different tissue compartments and stages of injury [200].

In addition to analyzing gene-expression levels, proteoform-aware methods increase the specificity of biomarkers by detecting PTMs, proteolysis products, and splice variants that may better distinguish between adaptive remodeling and maladaptive injury (e.g., phosphor-signaling intermediates, glycosylated adhesion molecules, or proteolytic fragments of ECM and metalloproteinase) [201,202]. In parallel, single-vesicle analysis using nanoscale flow cytometry and microfluidics provides single-particle resolution “liquid biopsies” of EVs by distinguishing vesicle origin and cargo (neural, astrocyte, and microglia) and tracking inflammasome- and pyroptosis-related signatures over time [203,204].

Together, these methods provide more mechanistically relevant sets of biomarkers that are aligned with the temporal progression from acute inflammation to chronic repair or secondary neurodegeneration [205]. Emerging system-level platforms, including dynamic contrast MRI and tracer-based clearance assays (glymphatic imaging), metabolomics, and human iPSC-derived neurovascular and organ-on-a-chip models, provide complementary approaches to measure clearance physiology, metabolic status, and ischemia/reperfusion-biomarker release in human-relevant conditions, thereby providing a basis for translational validation and therapeutic target identification [206,207].

Increased reliance on computational and artificial intelligence methods is being observed in order to convert these large numbers of data into clinically actionable biomarker profiles from the high-dimensional data generated by the aforementioned modalities [208]. Ischemic stroke research has been utilizing a variety of supervised machine-learning methods for the prediction of multi-modal biomarkers, including regularized logistic regression, Support Vector Machines (SVM), Random Forest, and Gradient Boosting (XGBoost/LightGBM), as well as several deep-learning architectures [209]. These deep-learning architectures include, but are not limited to, Convolutional Neural Networks (CNNs) and Encoder–Decoder Segmentation Models (U-net style models). In addition to the above applications, temporal models (i.e., Recurrent Neural Networks (RNNs), LSTMs, etc.) have also been utilized in modeling longitudinal biomarker trajectories, as well as the development of multimodal fusion methods (Late Fusion Ensembles and Attention-Based Methods), which can be used to combine imaging biomarkers, plasma/CSF biomarkers, clinical variables, and other data to develop composite predictors that identify stage-specific changes through the ischemic–reparative continuum [210].

### 7.2. Artificial Intelligence and Machine Learning in Biomarker Analysis

Data from a range of sources, such as poststroke biomarkers (multi-omics, extracellular vesicles, imaging, and clinical trajectories), are far too large to analyze in their entirety using traditional methods, thus driving the use of AI to analyze all of these modalities together and predict the outcomes of stroke [211,212].

Using supervised learning and deep multimodal fusion architectures will allow researchers to identify patterns in the data where there are interactions or overlap between different compartments (e.g., between the immune system, the blood vessels, the metabolism, etc.) and create new signatures that capture the interaction between these systems, leading to improved ability to stratify patients based on whether they have progressed to an infarct, developed a hemorrhage, suffered cognitive decline, or have achieved full recovery, as compared to analyzing each analyte individually [213,214].

Using dynamic predictions and/or state space models, researchers can now classify the patient into a set of “biological states” along the ischemia–repair spectrum (e.g., chronic inflammatory states driven by inflammasomes, reversible trophic states, degenerative axonal injury/complement activation states, etc.), thus enabling the clinician to intervene at a time that is biologically relevant to the specific pathophysiology present in the patient and thereby increasing the likelihood of a successful intervention [215]. The mechanistic interpretability of these predictive models may also be enhanced through the application of graph-based learning and Bayesian causal discovery to rank-order the most likely candidates involved in the disease process and to provide a directional relationship between the cellular programs. Radiotranscriptomics and imaging-omics approaches also enable the researcher to correlate MRI/PET-derived image features to molecular-activation states and therefore enable the development of cross-scale biomarkers that relate spatial tissue distribution to circulating biomarkers [216,217].

Future directions for research may include the development of generative trajectory models and “digital twin” models using variational autoencoders and transformer-based sequence models to predict how biomarkers evolve over time under different treatments [218] and federated learning to enhance external validity by allowing the model to be trained across multiple centers without requiring the central collection of all of the data [219].

A number of significant barriers to the translation of a model exist. In order to move a model forward into a clinical setting, there must first be a high level of agreement among clinicians regarding the standardization of many pre-analytic factors (sample type, sample timing, assays used, batch effects, and imaging protocols) [220]. Failure to achieve this level of standardization risks creating models that reflect differences between study sites rather than disease biology. Finally, adoption of any of these models will depend upon achieving adequate levels of interpretability (model explainability, calibration, and uncertainty quantification) and obtaining regulatory-grade evidence (including external validation, prospective longitudinal cohorts, standardization of results reporting, reproducibility of pipeline, and demonstration of clinical utility across diverse populations) [221].

### 7.3. Toward Predictive and Personalized Stroke Medicine

Predictive and personalized stroke medicine through advancements in AI and emerging technologies continues to move the field of stroke biomarkers from a static record of injury into an active and dynamic measure of prediction and precision therapy. Utilizing longitudinal data collection and analysis of molecular, imaging, and digital biomarkers enables the creation of an active record of the progression of stroke from inflammation to regeneration or impending degeneration, and informs trajectories and timing of therapeutic neuroprotection by biological readiness rather than chronological time points, thus increasing the potential for successful treatments [110].

The identification of molecular states via biomarkers creates a novel taxonomy for patient stratification and selection of precision therapy. For example, patients who have an abundance of inflammatory states (i.e., inflammasomes) will potentially respond to targeted inhibition of IL-1β or complement while patients who have an active and ongoing state of plasticity may benefit from neurotrophic factors, neuromodulation, or intensive rehabilitation. Conversely, patients moving toward degeneration may be eligible for senolytic, remyelination, or cognitive resilience interventions [222]. Molecular phenotyping in stroke has significant implications regarding how clinical trials will be designed, how cohorts will be created to identify therapeutic response, and how to avoid trial failure by providing therapies within a biologically relevant time frame. Digital biomarkers, such as wearable-device data on gait patterns, speech, and cognitive performance metrics, provide a singular source of information regarding a patient’s “real world” continuous presence. Utilizing subsequent longitudinal AI models linking gait and reaction-time data with cytokine and network connectivity will enable real-time sampling or changes in therapy based upon the evolving biological signature of the patient. Conversely, patients demonstrating trends toward plasticity microRNA levels will inform the need for early intensification of rehabilitation and establish tailored treatment downtime relative to their evolving biology [223].

Ultimately, these convergent technologies will lead to predictive stroke care defined by the multidimensional and dynamically evolving biomarker signatures of each individual patient. Unlike current stroke biomarkers, which are typically defined by a single molecule, the future biomarker of stroke will be a continually curated and dynamically evolving biomarked signature that is contextually relevant and informed, updated in real time, computationally processed, and results in a clinically meaningful response [22]. This will enable clinicians not only to understand what has occurred but also to predict what is likely to occur and to assess whether a targeted intervention can modify what might occur. This represents a paradigm shift in the way stroke is viewed as a dynamic biological process that can be tracked, predicted, and modified. Ultimately, it represents a new revolution of precision neurology based on the deep molecular logic of brain repair and recovery [224].

## 8. Conclusions and Future Perspectives

We believe that stroke biomarker science is currently experiencing a revolution. Biomarkers have gone from being static indicators of damage (or injury), by measuring the release of proteins after the death of cells or markers of damage to the blood–brain barrier, to being dynamic environmental biomarkers that illustrate how the body’s biology is undergoing change, and possibly even how to change the biology of the body. We attempted to discuss the transition of biomarkers, and how biomarkers can become not only post hoc reporters of what occurred to the brain but can also become pre hoc predictors of what is going to occur to the brain. This type of transition necessitates the integration and valuation of data across multiple scales. For example, a transcriptomics signature within the peri-infarct region is irrelevant without scaling to the spatial proteomics maps of angiogenesis or the vesicle proteomics and extracellular vesicle cargo signatures of glial transition, or the diffusion imaging findings of axonal remodeling. Additionally, combining gut metabolite analysis with microglial metabolomics and vagal nerve signaling can create a comprehensive view of the body’s systems’ communication regarding preparation of a complex trauma picture, while daily physical-activity data from wearable devices can add additional layers to a molecular systemic picture. Using such integrated biomarkers, system biomarkers, or signatures that indicate the overall status of the brain–body ecosystem, clinicians will be able to use these types of biomarkers to guide their practice, which would be predictive and mechanistically relevant to stroke.

We propose that clinical practice will be increasingly directed by such signals. Anti-inflammatory treatment will begin when the biomarkers demonstrate maladaptive immune patterns, rehabilitation will be matched to intervention at the time of maximal molecular changes that promote plasticity, and neurostimulation will be timed according to the time course of changes in network excitability. Digital twins—computer models that include genomic, proteomic, metabolic, imaging, and behavioral data—could allow clinicians to simulate disease trajectories, predict biological transitions, and tailor treatments to the changing status of each individual. Additionally, in research, using biomarker-defined states (e.g., inflammatory-persistent or plasticity-predominant) could improve study design, cohort diversity, and the ability to go beyond clinical endpoints to molecular inflection points. To achieve this vision, there will need to be increased collaboration across the world. Large, diverse datasets are required to develop predictive models, and federated learning networks could provide the necessary data while maintaining patient confidentiality and equity. Ethical issues must be treated with equal importance. Predictive biomarkers can reveal confidential information about individuals (e.g., risk of cognitive decline or probability of recovery) far in advance of when those events occur. Such information must be communicated with both humility and transparency, while respecting patient autonomy. Precision medicine must always remain probabilistic, never deterministic. Nonetheless, the trajectory appears to be positive. The tools to decipher the molecular language of the brain are now available, the methods to compute heterogeneous data are being rapidly developed, and the clinical imperative to transition from reactive to proactive care is greater than it has ever been before. We acknowledge that the current biomarkers—proteins, vesicles, and nucleic acids—are likely to be only a few words in a much larger language. As we continue to learn the language of the brain, we will be moving from describing the disease to working with the disease, and from being able to measure the degree of impairment to knowing how the brain wishes to repair itself.

The intent of this review was to make a contribution to the evolving story that continues to be written in laboratories and clinics, and by patients who may receive futures that we are learning about next. The most important question we perceive is not “what has happened?” but rather “what could potentially happen if we decide to actually listen to everything the injured brain is trying to communicate to us?”

## Figures and Tables

**Table 1:** Hyperacute ischemic stroke (minutes–hours): key molecular, cellular, and vascular mechanisms, associated biomarkers with temporal profiles, and clinical relevance (diagnosis, prognosis, and therapeutic targeting).

Process	Mechanistic Events	Key Biomarkers	Time Window	Clinical Significance	References
Metabolic collapse and excitotoxicity	Rapid ATP depletion halts Na^+^/K^+^ and Ca^2+^ pumps → ionic imbalance and depolarization → massive glutamate release → NMDA/AMPA overactivation → Ca^2+^ overload, ROS generation, and mitochondrial permeability transition → cytochrome c release and caspase activation.	Lactate, succinate, glutamate, aspartate, NSE, UCH-L1, SBDPs, 8-OHdG, MDA, cytochrome c, caspase-3, and HIF-1α.	0–2 h	Define ischemic-core formation. Succinate accumulation predicts ROS burst on reperfusion. NSE and UCH-L1 correlate with infarct size and outcomes.	[54,55]
Purine catabolism and stress response	ATP-breakdown products (hypoxanthine, and xanthine) rise; immune cells upregulate stress-responsive transcription factors and cytokine genes.	Hypoxanthine, xanthine, ATF3, NF-κB, and IL-6 mRNA.	0.5–3 h	Reflect energy failure and systemic immune priming; correlate with stroke severity.	[56,57]
miRNA release	Neuronal and glial release of regulatory miRNAs modulate apoptosis, excitotoxicity, and inflammation.	miR-124, miR-9, miR-21, and miR-210.	1–4 h	Predicts infarct expansion and early injury; enables rapid blood-based diagnosis.	[58,59,60]
BBB breakdown and endothelial activation	ROS, cytokines, and proteases (MMP-2/9) degrade tight junctions (claudin-5 and occludin); endothelial cells shed adhesion molecules; and thrombogenic activation ensues.	MMP-2, MMP-9, sICAM-1, sVCAM-1, sE-selectin, vWF, and TJ fragments.	1–6 h	MMP-9 predicts infarct size and hemorrhagic transformation risk. Adhesion markers reflect endothelial activation and leukocyte recruitment.	[61,62,63]
Astrocyte and pericyte response	Astrocytes swell via AQP4 and release GFAP and S100B; pericytes detach, releasing PDGFR-β; and angiopoietins and ephrins alter perivascular signaling.	GFAP, S100B, PDGFR-β, Ang-1/2, and EphB ligands.	2–6 h	GFAP differentiates ischemic vs. hemorrhagic stroke. PDGFR-β indicates microvascular destabilization.	[64,65]
Neurovascular unit leakage	BBB breach permits CNS molecules and exosomes into blood; and systemic cytokines and leukocytes exacerbate injury.	NfL, GFAP, and neuronal exosomes.	2–6 h	Blood-based surrogates of CNS damage; useful for real-time monitoring of BBB integrity.	[66,67]
Extracellular vesicles	Neurons, glia, and endothelial cells release EVs with dynamic cargo reflecting cell stress and state.	EV-synaptophysin, EV-GFAP, miR-124, and miR-9.	1–6 h	Offer minimally invasive biomarkers; support time-resolved profiling and personalized monitoring.	[68,69]
Imaging correlates	Perfusion MRI maps hypoperfusion and penumbra; DWI detects cytotoxic edema; and ADC and Tmax assess microvascular status.	ADC, Tmax, and perfusion maps.	Minutes–6 h	Complement molecular biomarkers; refine diagnosis, prognosis, and therapeutic targeting.	[70]
Composite biomarker panels and AI	Multi-omics and imaging data integrated by ML for real-time classification and prediction.	Composite panels and AI classifiers.	≤6 h	Improve diagnostic precision, predict hemorrhagic risk; guide reperfusion and neuroprotective strategies.	[71,72]

**Table 2:** Chronic ischemic stroke (weeks–months): key biomarker signatures reflecting recovery and degeneration, spanning synaptic and myelin remodeling, persistent inflammation, glial senescence, and secondary neurodegeneration.

Domain	Key Processes	Representative Biomarkers	Temporal Dynamics	Clinical/Translational Significance	References
Structural and network plasticity	Axonal sprouting, synaptic remodeling, dendritic spine turnover, and network reorganization.	GAP-43, SPRR1A, βIII-tubulin, BDNF, NGF, PSD-95, Homer, CREB, and CaMKII.	Weeks–months, peaking during rehabilitation and task engagement.	Indicators of neuroplastic potential and recovery trajectory; correlate with motor and cognitive gains; and guide intensity and timing of rehabilitation.	[159,160]
Myelin remodeling	Oligodendrocyte proliferation and differentiation, remyelination, and conduction restoration.	Olig2, Sox10, PDGF-AA, MBP, PLP, MOG, and oligodendrocyte-derived EVs.	Weeks–months, often delayed vs. synaptic changes.	Reflects white-matter repair and conduction recovery; predicts long-term connectivity and functional outcomes.	[161,162]
Epigenetic and transcriptomic remodeling	DNA methylation, histone acetylation, chromatin reorganization, and lncRNA/circRNA regulation.	Bdnf promoter methylation, H3K27ac, lncRNA MALAT1, circHIPK3, and miR-132.	Persistent, weeks–months, and dynamic with rehabilitation.	Mark latent plasticity potential; may stratify patients for epigenetic therapies or delayed rehab responsiveness.	[163,164]
Glial activation and maladaptive remodeling	Chronic astrocyte reactivity, inhibitory ECM formation, microglial activation, and complement overactivation.	GFAP, vimentin, CSPGs (neurocan and brevican), IL-1β, TNF-α, sTREM2, sCD14, TSPO-PET, C1q, and C3.	Persistent, weeks–months, and often plateauing if untreated.	Markers of maladaptive scarring and neuroinflammation; used to predict cognitive decline and network rigidity.	[165,166]
Senescence and chronic inflammation	Glial senescence, sustained inflammasome activation, and systemic immune dysregulation.	p16^INK4a, p21, IL-6, GDF15, HMGB1, NLRP3, Th17/Treg ratio, IL-17A, and sIL-2R.	Persistent, weeks–months, and associated with poor recovery trajectories.	Stratify risk of cognitive deterioration; potential targets for late-phase immunomodulation or senolytic therapies.	[167,168]
Secondary neurodegeneration	Wallerian degeneration, trans-synaptic spread, and delayed neuronal loss.	NfL, myelin-breakdown products, phosphorylated tau, Aβ42/40 ratio, and neurogranin.	Peaks weeks–months; may persist chronically.	Predicts progressive structural and cognitive decline; overlaps with neurodegenerative signatures and dementia risk.	[169,170]
Precision therapeutic windows	Composite biomarker panels, epigenetic clocks, and multi-omics state classification.	Multi-omics profiles (proteomic, transcriptomic, and metabolomic), CSPG-EVs, neurogenic miRNAs, and digital biomarkers.	Dynamic, longitudinal; inform evolving biological states.	Identify optimal therapy timing, guide intensity and modality selection, and support adaptive and personalized interventions.	[171,172]

## Data Availability

The data presented in this study are available upon request from the corresponding author.
